# A mixed treatment comparison to compare the efficacy and safety of botulinum toxin treatments for cervical dystonia

**DOI:** 10.1007/s00415-016-8050-2

**Published:** 2016-02-25

**Authors:** Yi Han, Andrea L. Stevens, Khashayar Dashtipour, Robert A. Hauser, Zoltan Mari

**Affiliations:** WG Consulting, 200 Fifth Avenue, New York, NY 10010 USA; Faculty of Medical Offices, School of Medicine, Loma Linda University, 11370 Anderson, Suite B-100, Loma Linda, CA 92354 USA; Health Byrd Institute, University of South Florida, 4001 E. Fletcher Ave, 6th Floor, Tampa, FL 33613 USA; School of Medicine, Johns Hopkins University, 600 N. Wolfe Street, Meyer 6-181B, Baltimore, MD 21287 USA

**Keywords:** Cervical dystonia, Botulinum toxin, TWSTRS, Mixed treatment comparison

## Abstract

A systematic pair-wise comparison of all available botulinum toxin serotype A and B treatments for cervical dystonia (CD) was conducted, as direct head-to-head clinical trial comparisons are lacking. Five botulinum toxin products: Dysport^®^ (abobotulinumtoxinA), Botox^®^ (onabotulinumtoxinA), Xeomin^®^ (incobotulinumtoxinA), Prosigne^®^ (Chinese botulinum toxin serotype A) and Myobloc^®^ (rimabotulinumtoxinB) have demonstrated efficacy for managing CD. A pair-wise efficacy and safety comparison was performed for all toxins based on literature-reported clinical outcomes. Multi-armed randomized controlled trials (RCTs) were identified for inclusion using a systematic literature review, and assessed for comparability based on patient population and efficacy outcome measures. The Toronto Western Spasmodic Torticollis Rating Scale (TWSTRS) was selected as the efficacy outcome measurement for assessment. A mixed treatment comparison (MTC) was conducted using a Bayesian hierarchical model allowing indirect comparison of the interventions. Due to the limitation of available clinical data, this study only investigated the main effect of toxin treatments without explicitly considering potential confounding factors such as gender and formulation differences. There was reasonable agreement between the number of unconstrained data points, residual deviance and pair-wise results. This research suggests that all botulinum toxin serotype A and serotype B treatments were effective compared to placebo in treating CD, with the exception of Prosigne. Based on this MTC analysis, there is no significant efficacy difference between Dysport, Botox, Xeomin and Myobloc at week four post injection. Of the adverse events measured, neither dysphagia nor injection site pain was significantly greater in the treatment or placebo groups.

## Introduction

Cervical dystonia (CD), formerly referred to as spasmodic torticollis, is a condition characterized by simultaneous and sustained contractions of both agonist and antagonist muscles of the neck [1]. The majority of patients complain of pain, which is not a common feature of other focal dystonias [1]. Head rotation (torticollis) is common, but head tilt (laterocollis), neck extension (retrocollis) and flexion (anterocollis) may also occur, often in combination [2]. CD is the most common type of focal dystonia encountered in neurological practice, with an estimated prevalence of 57 per million in Europe [3], or as much as 0.4 % of the total population of the United States [4–6]. While there is a need for more accurate population-based epidemiology studies of CD, this prevalence rate was confirmed by Defazio et al. in a 2012 review article [7].

Botulinum toxin (BoNT), a neurotoxin produced by the bacterium* Clostridium botulinum*, causes impairment of neuromuscular transmission leading to flaccid paralysis [8]. Intramuscular injections of BoNT have been shown to be efficacious and well tolerated when used to treat CD [9–12], and are therefore recommended as first-line therapy by current treatment guidelines [13, 14].

Two distinct serotypes of BoNT are available in clinical practice, and currently various different formulations of serotypes A and B are being used for the treatment of dystonia. Five BoNT products are available in various countries for the management of CD; Dysport^®^ (abobotulinumtoxinA), Botox ^®^ (onabotulinumtoxinA), Xeomin^®^ (incobotulinumtoxinA), Prosigne^®^ (Chinese botulinum toxin serotype A) and Myobloc^®^ (rimabotulinumtoxinB) [15]; however, there are limited data available to show a head-to-head comparison of these treatments in randomized clinical trials (RCT). It is unrealistic to expect that head-to-head RCT data will be available for all treatments, as clinical trials are expensive to conduct. In addition, the clinical and scientific values of such direct pair-wise comparisons are often questioned, due to trial design issues, and companies are reluctant to fund head-to-head comparisons of products likely to show similar efficacy. Importantly, with BoNT treatment of CD, there is a dosing comparability issue that is not easily resolved [16].

Placebo-controlled studies have investigated the efficacy and safety of BoNTs, demonstrating a significant improvement from baseline in outcome scores for all treatments of interest, with similar safety profiles [10–12, 16–19]. Equivalent efficacy of Dysport to Botox has been demonstrated directly in two multicenter, double-blind, randomized studies in blepharospasm and CD, respectively [9, 20, 21]. Head-to-head comparisons have also demonstrated Myobloc to be non-inferior to Botox [16, 19], and Prosigne to have equivalent efficacy, safety, and tolerability as Botox [22]. Data from placebo controlled trials utilizing different treatments can be used in statistical analyses, allowing the data to be combined to compare and contrast their efficacy and safety benefits.

Meta-analysis is a statistical approach where direct clinical data from multiple sources can be combined to compare the efficacy and safety of two treatments [23], where

- For a large proportion of health interventions, there is no direct evidence that relates the interventions to the health outcome [24],
- Direct information exists on a specific treatment comparison, but does not provide enough information for a substantial statistical analysis. We then need to ‘borrow strength’ from indirect comparisons [25],
- No single treatment comparison is of specific interest; instead there is a need to simultaneously compare [26], or even rank, several treatments [27, 28].

The mixed treatment approach extends traditional meta-analysis to include indirect comparisons using hierarchical Bayesian methods, meaning multiple treatments can be compared in a single analysis [23]. This allows the efficacy and safety outcomes of numerous RCTs for multiple treatments to be compared head to head, potentially negating the need for additional RCTs and the associated complications and expense. This research was conducted to provide a systematic pair-wise comparison of all available BoNT serotype A and B treatments for CD, in light of the lack of direct head to head clinical trial data. A pair-wise efficacy comparison was performed for Dysport, Botox, Xeomin, Prosigne and Myobloc based on literature-reported clinical outcomes.

## Methods

### Search strategy and selection criteria

A search for multi-armed randomized controlled trials (RCTs) involving Dysport, Botox, Xeomin, Prosigne and Myobloc was conducted using several databases (Embase, Medline and Medline (R) In-Process). Each database was searched from inception to February 2014 and with no restriction on the language of the papers. The search was kept particularly broad with search terms on Botulinum toxin A, Botulinum toxin B, CD, cervical dystonia, TWSTRS, Toronto Western Spasmodic Torticollis Rating Scale, and a filter for randomized controlled trials was used in order to increase sensitivity.

### Inclusion criteria

Only full-published reports of RCTs including patients affected by CD were considered; letters and abstracts were excluded. Only RCTs with TWSTRS measured as primary or secondary endpoints were included.

### Exclusion criteria

RCTs studying disease areas other than CD or having as primary or secondary endpoint measures other than TWSTRS were excluded. Also studies comparing other interventions in CD were excluded.

### Interventions

The intervention of at least one study group included one of the following drugs and dosing regimens in clinical use: Dysport, Botox, Xeomin, Prosigne and Myobloc.

### Efficacy outcome measures

The Toronto Western Spasmodic Torticollis Rating Scale (TWSTRS) total score was selected as the efficacy outcome measurement for assessment [29]. The TWSTRS is a validated CD scale that captures the clinical features of CD, and includes a videotape protocol such that all patients are viewed in a standardized fashion [29]. The TWSTRS is comprised of 3 subscales: severity, disability, and pain, each of which is scored independently. The total of these three comprises the TWSTRS total score, which is scored from 0–87 (best to worst). It is worth mentioning that the Tsui score is also used in some clinical studies. However, the Tsui score is not validated in the same manner as the TWSTRS, and its relative simplicity means that several features of CD, such as pain or disability, are not covered. There are also no clear criterion definitions for the Tsui score ratings of “mild”, “moderate” and “severe” [30]. These factors make the Tsui scale unsuitable for MTC analysis.

### Safety outcome measure

BoNT products are well tolerated and safe with proper injection into a target site. Common adverse effects (>10 % frequency) include excessive muscle weakness, injection site pain, dry mouth, dysphagia, and fatigue. From a clinical perspective, the two most common clinically relevant adverse events, due to BoNT injections, are related to unwanted weakness. Injecting too much toxin in a particular area of the neck can cause weakness, leading to abnormal head positions or limitations in neck range of motion. Moreover, toxins can diffuse beyond the confines of injected muscles, potentially causing dysphagia due to weakness of swallowing muscles, especially when anterior neck muscles are injected [31]. Dysphagia can be uncomfortable, may necessitate a change in diet, and can lead to aspiration. Another common adverse event is injection site pain. This is usually transient and resolves in minutes to days. Many factors can potentially contribute to injection site pain including injection technique, type and size of needle used, volume and location of injection, and make-up and pH of the toxin and its associated constituents [32, 33]. To aid healthcare professionals in the correct evaluation of safety profiles for different toxin products, data was collected from these clinical studies for all reported major adverse events.

### Statistical analysis

**Fig. 1:**
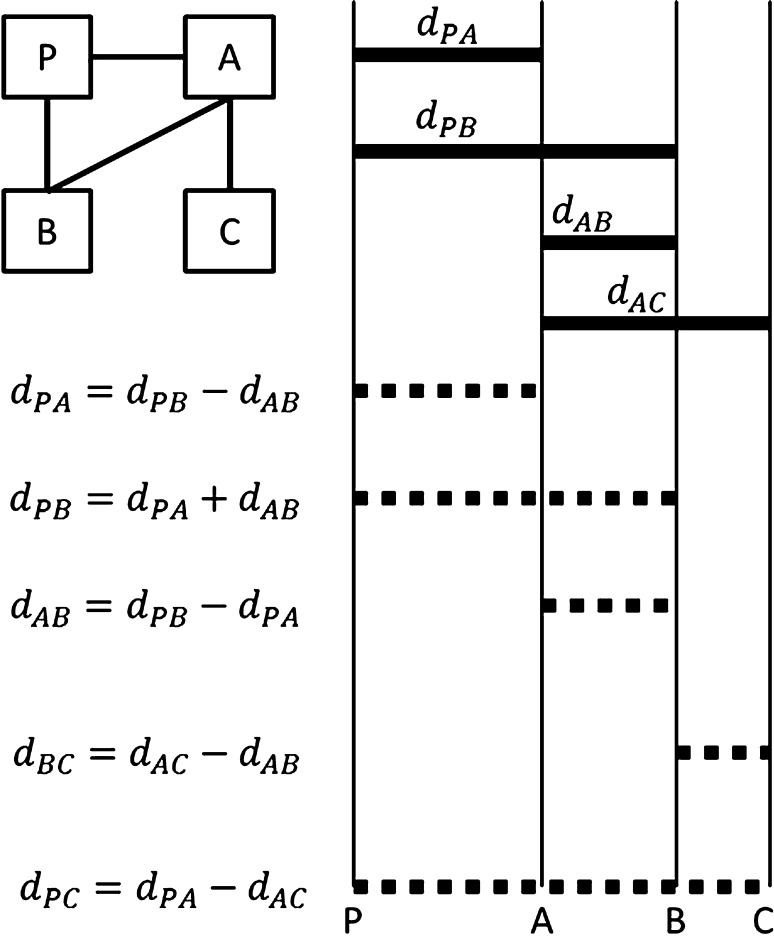
Diagram representing the network of studies reflecting mixed treatment comparisons of PA trials, PB trials, AB trials, and AC trials (adapted from Jansen) [23]

A mixed treatment comparison (MTC) was conducted using a Bayesian hierarchical model allowing indirect comparison of the efficacies of the interventions. MTC is a generalization of standard meta-analysis for pair-wise trials, to a simultaneous analysis of multiple pair-wise comparisons [34], for example, interventions A, B and C. Given the network of direct comparisons across the range of interventions, indirect estimates can be obtained for *d*_PA_, *d*_PB_, *d*_AB_, *d*_BC_, and *d*_PC_ (Fig. 1) where P stands for placebo. Given the mathematical relationships between the true underlying estimates of the different comparisons in the network, we have both direct and indirect evidence available for all the pair-wise comparisons, except for the BC comparisons (only indirect evidence) and the AC comparisons (only direct evidence). Hence, the advantages of the simultaneous analysis with MTC are that (1) estimates for indirect comparisons are obtained, and (2) indirect comparisons can support evidence for direct estimates [35].

To explicitly account for effects due to heterogeneity from different trials, a random effect MTC method was used incorporating the following formula:

Let *y*_*ij*_ be the observed efficacy of treatment *j* in the *i* th study. It can be considered as a random observation from normal distribution centered at *Y*_*ij*_ and *Y*_*ij*_ be the unobserved mean efficacy with variance *σ*_*ij*_. *Y*_*ij*_ can be further expressed as the sum of baseline treatment effect μ_*ib*_ and efficacy differential *δ*_*ijb*_. Study level efficacy differential is a random outcome drawn from normal distribution centered at true efficacy differential Δ_*jb*_. From coherence assumption, pair-wised efficacy differential can be estimated indirectly through Δ.

$$ y_{ij} \sim N(Y_{ij} , \sigma_{ij}  ) $$

$$ Y_{ij} = \mu_{ib} + \delta_{ijb}   (j \ne b) $$

$$ \delta_{ijb} \sim N(\Delta_{jb} , \sigma ) $$

$$ \Delta_{jk} = \Delta_{jb}  {-} \Delta_{kb} (j,k \ne b) $$

Adverse events were modeled as a binominal distribution where the number of patients with a specific adverse event in trial *i* treated with toxin *j* is defined by *p*_*ij*_ and *N*_*i*_, the rate of adverse event and sample size for treatment *j* in trial *i*:

$$ n_{ij} = {\text{Bin}}(p_{ij} , N_{i} ) $$

Logit transformation of *p*_*ij*_ will be treated as a random variable with normal distribution. Relative safety against placebo was measured using the logarithm of odds ratio (LOR), where a positive number represents an increased risk.

## Results

**Fig. 2:**
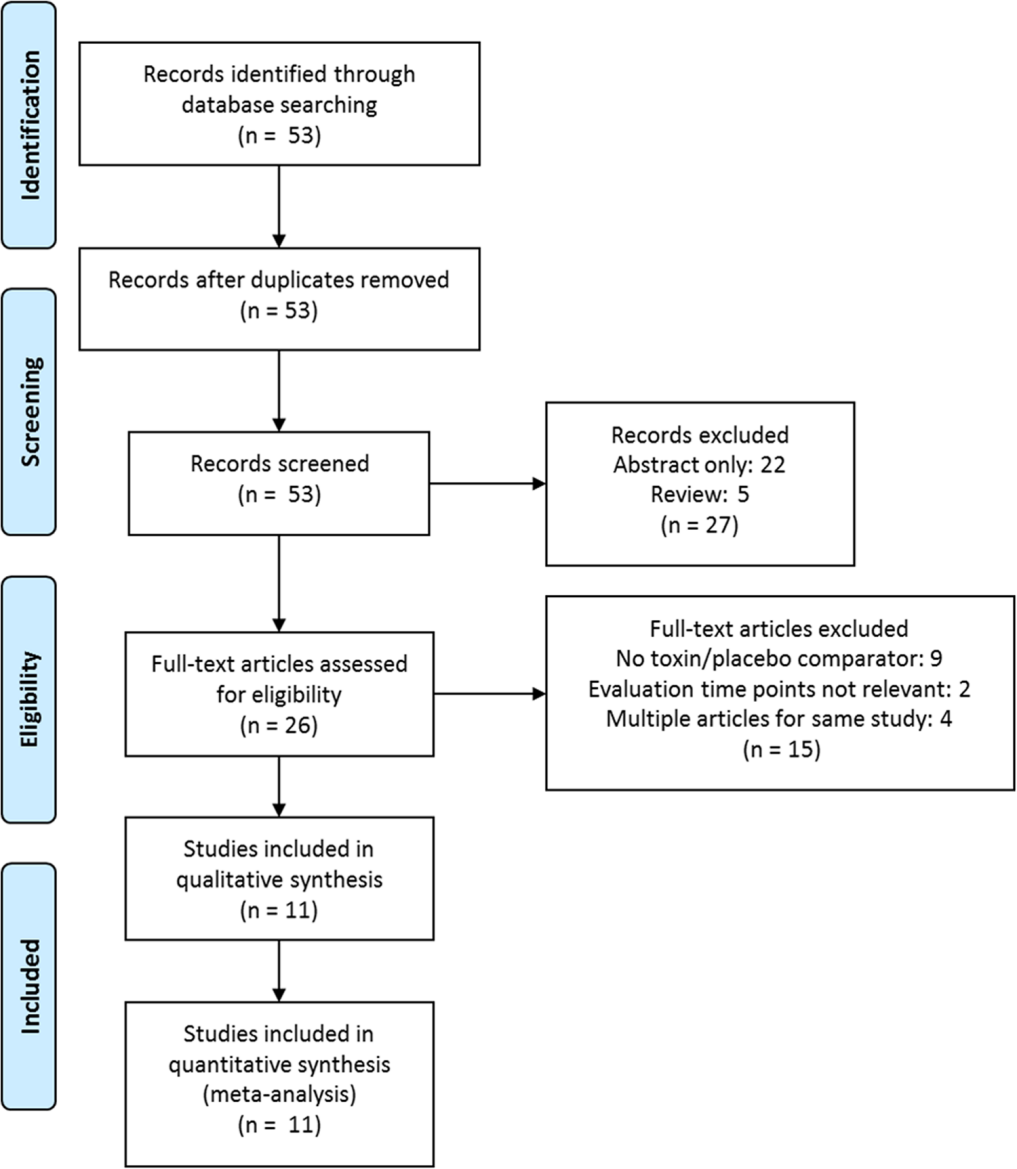
MTC PRISMA Flow Diagram (adapted from Moher et al.) [36]

A total of 11 RCTs were identified through a systematic literature review carried out according to PRISMA (preferred reporting items for systematic reviews and meta-analyses) guidelines [36], providing data on 1295 participants. The process for selection and exclusion of studies is detailed in Fig. 2.

Table 1 summarizes the study characteristics of the trials included in the analysis.

**Table 1:** Characteristics of included studies

Publications	Interventions	Endpoints	Study design	Number randomized	Treated patients
Comella J Neurological Sciences 2011	Xeomin 120 U Xeomin 240 U	Change from baseline to week 4 on the TWSTRS total score	Prospective, double-blind, randomized, placebo-controlled, multicenter clinical trial in botulinum toxin-treated or toxin-naïve CD patients	233	78 81
Truong Movement Disorders 2005	Dysport 500 U	Change in TWSTRS total score at week 4 compared with baseline	Prospective, double-blind, randomized, placebo-controlled, multicenter clinical trial	80	37
Truong Parkinsonism and Related Disorders 2010	Dysport 500 U	Change from baseline in the TWSTRS severity, disability, and pain subscale scores at week 4 after the start of each treatment cycle	Randomized, double-blind study to investigate the efficacy and safety of intramuscular admin-istration of Dysport compared to placebo for the treatment of CD	116	55
Ranoux JNNP 2002	Botox 100 U Dysport 500 U	Change in the TWSTRS pain scale score between baseline and control visit	Double blind, randomised, three period cross over study	54	51
Quagliato Clinical Neuropharmacology 2010	Botox 100 U Prosigne 100 U	Change of the TWSTRS scores between the baseline and control visits	Prospective, randomized, double-blind study to compare Botox and Prosigne in the treatment of cervical dystonia	24	24
Kaji Brain and Nerve 2013	Myobloc 2500 U Myobloc 5000 U Myobloc 10,000 U	Change in TWSTRS total score at 4 weeks post dose from baseline	Single-dose, placebo-controlled, double-blind, dose–response study of NerBloc^®^(Myobloc) in patients with cervical dystonia	133	98
Brashear Neurology 1999	Myobloc 5,000 U Myobloc 10,000 U	TWSTRS total score at week 4	16-week, randomized, multicenter, double-blind, placebo-controlled trail in type A-responsive patients with CD	109	36 37
Brin Neurology 1999	Myobloc 10,000 U	TWSTRS total score at week 4	16-week, double-blind, placebo-controlled trial of BoNT/B in type A-resistant patients with CD	77	39
Pappert Movement Disorders 2008	Botox 150 U Myobloc 10,000 U	Change in TWSTRS from baseline to week 4 post-injection	International, multi-center, double-blind, randomized, comparator study	111	55 56
Comella Neurology 2005	Botox 250 U Myobloc 10,000 U	Change in total TWSTRS score and change in subscale TWSTRS scores at maximal efficacy (week 4)	Randomized, double-blind, parallel-arm study	139	74 65
Lew Neurology 1997	Myobloc 2,500 U Myobloc 5,000 U Myobloc 10,000 U	TWSTRS-total score at 4 weeks following study drug administration	Randomized, multicenter, double-blind, placebo-controlled, four-arm, parallel-group outpatient study	122	31 31 30

**Fig. 3:**
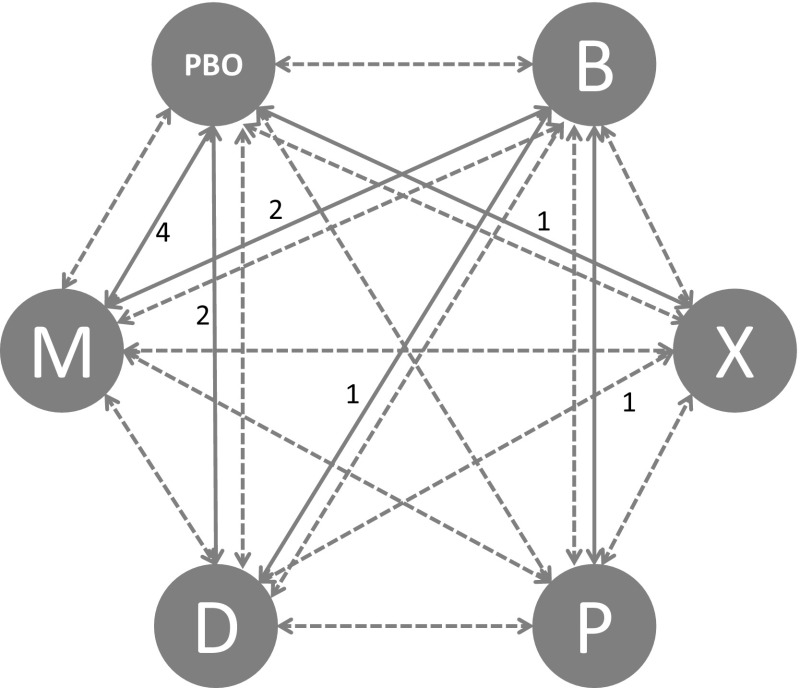
TWSTRS PAIN and dysphagia network (*PBO* placebo, *B* botox, *X* xeomin, *P* prosigne, *D* dysport and *M* myobloc)

**Fig. 4:**
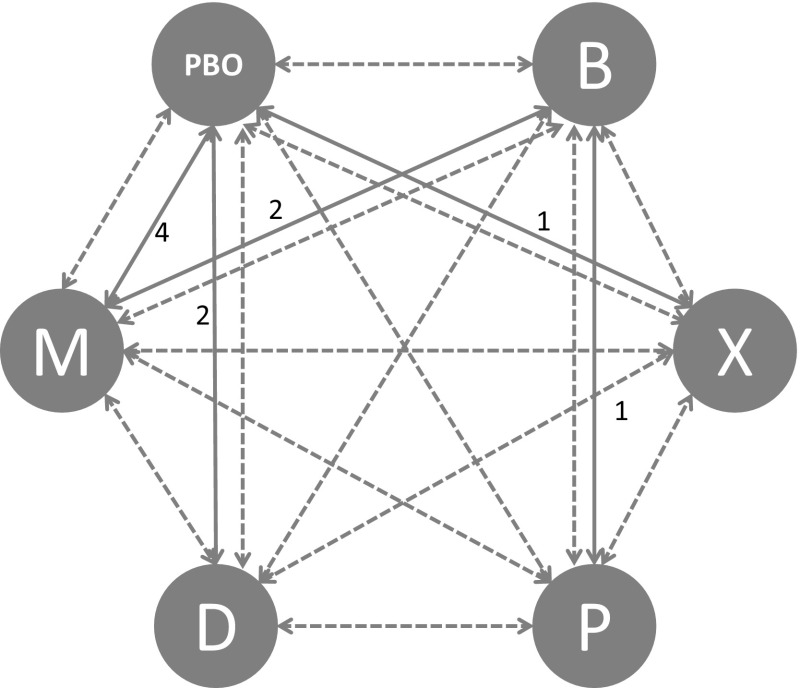
TWSTRS total, disability and severity network (*PBO* placebo, *B* botox, *X* xeomin, *P* prosigne, *D* dysport, *M* myobloc)

**Fig. 5:**
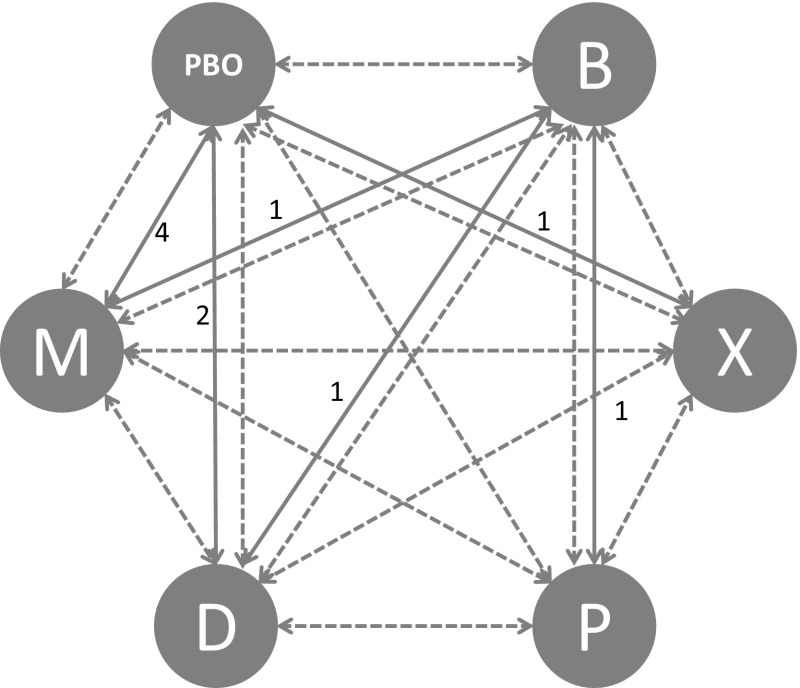
Injection site pain network (*PBO* placebo, *B* botox, *X* xeomin, *P* prosigne, *D* dysport, *M* myobloc)

Four trials compared Myobloc vs. placebo, two trials compared Dysport vs. placebo, one trial compared Xeomin vs. placebo, one trial compared Dysport vs. Botox, one trial compared Prosigne vs. Botox and two trials compared Myobloc vs. Botox. In this study, we focused our investigation on efficacy measured by the change in TWSTRS score 4 weeks post injection time. If standard deviations for week four TWSTRS score changes were not reported for a trial arm, the largest reported standard deviation of baseline or week four in that arm was used. For studies which only reported median and range values, mean and standard deviation was calculated based on the methods described in Hozo et al. [37]. In cases where no variance information was disclosed, the largest variances within the selected studies were used by default. There was reasonable agreement between the number of unconstrained data points, residual deviance and pair-wise results, suggesting a coherent network. The network of studies for each efficacy and safety outcome measure is shown in Figs. 3, 4 and 5. Numbers correspond to the number of studies compared within each part of the network.

**Fig. 6:**
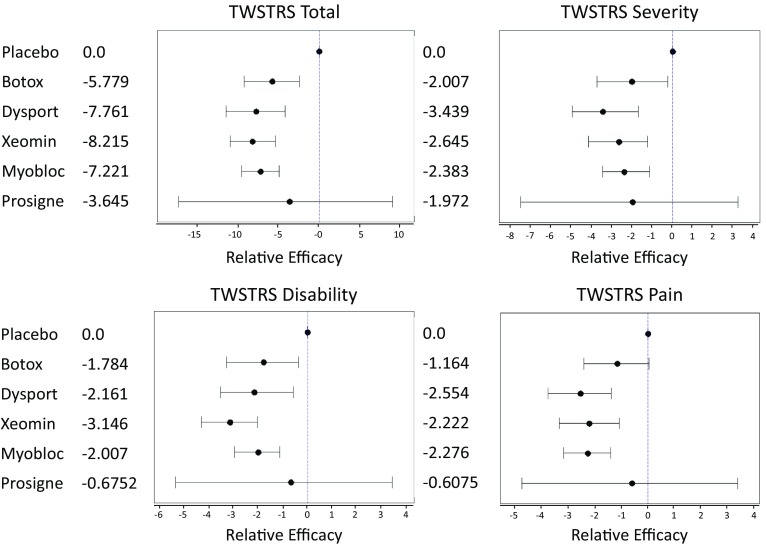
Forest plots detailing the efficacy results of the MTC

The results of the MTC are shown in Table 2 and Fig. 6.

**Table 2:** Relative efficacy measured by median TWSTRS subscale score 4 weeks post injection

Change at 4 weeks	TWSTRS total		TWSTRS severity		TWSTRS disability		TWSTRS pain	
BoNT	Median	95 % CI	Median	95 % CI	Median	95 % CI	Median	95 % CI
Botox	−5.779	−9.222, −2.399	−2.007	−3.726, −0.2261	−1.784	−3.293, −0.3679	−1.164	−2.419, 0.0401
Dysport	−7.761	−11.43, −4.195	−3.439	−4.938, −1.687	−2.161	−3.536, −0.5743	−2.554	−3.777, −1.392
Xeomin	−8.215	−10.97, −5.352	−2.645	−4.133, −1.219	−3.146	−4.318, −2.029	−2.222	−3.36, −1.084
Myobloc	−7.221	−9.535, −4.91	−2.383	−3.451, −1.138	−2.007	−2.962, −1.119	−2.276	−3.184, −1.408
Prosigne	−3.645	−17.31, 9.059	−1.972	−7.483, 3.23	−0.6752	−5.357, 3.448	−0.6075	−4.761, 3.393

As shown in Table 2, all toxin treatments, apart from Prosigne, demonstrated a similar range of efficacy relative to placebo. Excluding Prosigne, the median TWSTRS total score improvements over placebo are within a range of (−5.78, −8.22), with the sub-scale efficacy ranges being even narrower, as expected. The trends of efficacy measures are consistent with product label information and FDA marketing authorization. The median and 95 % confidence interval for each toxin are shown in the forest plots (Fig. 6).

MTC analysis clearly demonstrated that BoNTs Dysport, Botox, Xeomin and Myobloc are more efficacious in managing CD when compared with placebo. However, there is no statistically significant performance difference among these toxins. Prosigne efficacy could not be confirmed because the data was extracted from a single study in which the outcomes deviated significantly compared with other published work.

**Fig. 7:**
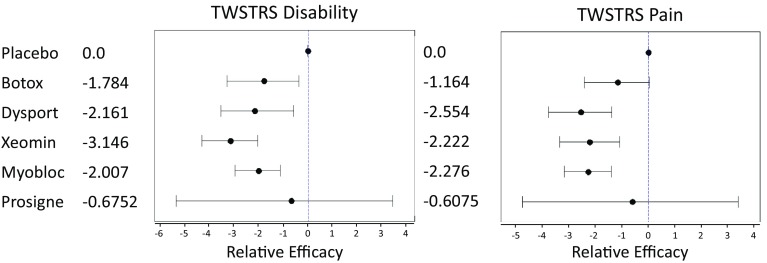
Forest plots detailing the MTC results for adverse events dysphagia and injection site pain

Safety data reporting is not systematic in the public domain. Within the included journal articles from this systematic literature review, a sufficient number of studies were identified with data supporting analysis on the adverse events dysphagia and injection site pain. The analysis results are shown in Table 3, with forest plots to graphically depict the median Log Odds Ratio (LOR) and its 95 % confidence interval (Fig. 7).

**Table 3:** Adverse event MTC results

Log odds ratio (LOR)	Dysphagia			Injection site pain		
	Median	95 % CI low	95 % CI high	Median	95 % CI low	95 % CI high
Botox	1.012	−0.3997	2.855	1.076	−0.6695	3.065
Dysport	2.212	0.8621	4.108	0.9522	−0.01974	2.016
Xeomin	2.086	0.347	4.349	0.1427	−1.123	1.611
Myobloc	2.144	1.116	3.818	0.2664	−0.5163	1.027
Prosigne	1.293	−1.264	4.366	−2.238	−5.726	1.417

Incidence of dysphagia and injection site pain measured at 4 weeks post injection

Similar to the trends observed in the efficacy comparison, the Prosigne data showed too much variance to draw reliable conclusions. For the other toxins included in this study, all 95 % confidence intervals were overlapping. This is a clear indication that these toxins do not have a statistically significant different incidence rate for the adverse events under investigation; dysphagia and injection site pain. It is also interesting to see that for injection site pain, the rates of adverse events from toxin treatment were not different from placebo at the 95 % confidence level.

## Discussion

This is the first reported mixed treatment comparison of five BoNT treatments: Dysport, Botox, Xeomin, Myobloc and Prosigne, for the treatment of CD. Based on the comprehensive systematic literature review and Bayesian hierarchical model mixed treatment comparison, this research suggests that BoNT treatments Dysport, Botox, Xeomin and Myobloc were effective compared to placebo in the treatment of CD as measured by the Toronto Western Spasmodic Torticollis Rating Scale total score at week four post-injection. Moreover, based on this MTC analysis, there is no significant efficacy difference between Dysport, Botox, Xeomin and Myobloc at week four post-injection. Prosigne data was inconsistent with other evidence included in this analysis. It is not possible to draw meaningful conclusions for the efficacy of Prosigne given the large variance of the model estimates. The advantage of this Bayesian MTC method, in comparison with traditional pair-wise meta-analyses, is that a larger range of data is taken into account in one single analysis. Moreover, the MTC approach includes the ability to compute the relative efficacy of each treatment without breaking trial randomization, which is a key factor when comparing several interventions [23].

Dysphagia and injection site pain were the most frequently reported adverse events seen across all studies included in the analysis. This was expected as both are known side effects of treatment with BoNT. Dysport, Botox, Xeomin and Myobloc did not demonstrate a statistically significant different incidence rate for the adverse events under investigation. Previous studies have demonstrated an increased occurrence of dysphagia with BoNT serotype B compared with serotype A in the treatment of CD (*p* = 0.0005) [16], but this was not demonstrated in the MTC.

Overall, the results of this study suggest that both BoNT serotype A and botulinum serotype B treatments for CD provide a similar efficacy to one another at week four, and all but Prosigne are effective compared with placebo. Of the adverse events measured, neither dysphagia nor injection site pain were significantly greater in the treatment or placebo groups.

This MTC demonstrated an extension of traditional meta-analysis by including multiple different pair-wise comparisons across a range of different interventions. The advantages of Bayesian MTC include the comparison of drugs in the absence of head-to-head data; probability statements that one drug is better (e.g., more efficacious, safer) than another or not; and probability calculations that one drug is best (rank-order the interventions) or that all are similar. Hence, MTCs can provide useful information for (medical) decision-making.

### Study limitations

This analysis was based on published clinical results in the public domain. Conclusions from this study are subject to publication bias which may exist in pharmaceutical clinical research. Furthermore, due to the limited number of available trials, this study only compared the main effects of exposure to different treatments. Other contributing factors, such as dosing, formulation and patient characteristics cannot be explored explicitly. There is no uniformly accepted dosing conversion ratio between the different toxins. Large well-designed head-to-head clinical trials are needed to generate reliable information for patients switching between these treatments. Our analysis used a random effect method to compensate for these confounders. Adverse events investigated in this study were selected based on the availability of published data. Some other interesting safety events, such as neck weakness, were not included due to insufficient data.
